# Slab melting boosts the mantle wedge contribution to Li-rich magmas

**DOI:** 10.1038/s41598-024-66174-y

**Published:** 2024-07-02

**Authors:** Erwin Schettino, Igór González-Pérez, Claudio Marchesi, José María González-Jiménez, Michel Grégoire, Romain Tilhac, Fernando Gervilla, Idael F. Blanco-Quintero, Alexandre Corgne, Manuel E. Schilling

**Affiliations:** 1 Department of Earth Sciences, ETH Zürich, Clausiusstrasse 25, 8092 Zürich, Switzerland; 2 Instituto Andaluz de Ciencias de La Tierra (IACT), Consejo Superior de Investigaciones Científicas-Universidad de Granada, Avenida de Las Palmeras 4, 18100 Armilla, Spain; 3 Departamento de Mineralogía y Petrología, Universidad de Granada, Avenida Fuentenueva S/N, 18002 Granada, Spain; 4 Géosciences Environnement Toulouse (GET), Observatoire Midi Pyrénées, CNRS, CNES-IRD-Université Toulouse III, 14 Av. E. Belin, 31400 Toulouse, France; 5 Departamento de Ciencias de La Tierra y del Medio Ambiente, Facultad de Ciencias, Universidad de Alicante, 03690 Alicante, Spain; 6 Instituto de Ciencias de La Tierra, Facultad de Ciencias, Universidad Austral de Chile, 5090000 Valdivia, Chile

**Keywords:** Geochemistry, Geodynamics, Mineralogy, Petrology

## Abstract

The lithium cycling in the supra-subduction mantle wedge is crucial for understanding the generation of Li-rich magmas that may potentially source ore deposition in continental arcs. Here, we look from the mantle source perspective at the geological processes controlling the Li mobility in convergent margins, by characterizing a set of sub-arc mantle xenoliths from the southern Andes (Coyhaique, western Patagonia). The mineral trace element signatures and oxygen fugacity estimates (FMQ >  + 3) in some of these peridotite xenoliths record the interaction with arc magmas enriched in fluid-mobile elements originally scavenged by slab dehydration. This subduction-related metasomatism was poorly effective on enhancing the Li inventory of the sub-arc lithospheric mantle, underpinning the inefficiency of slab-derived fluids on mobilizing Li through the mantle wedge. However, major and trace element compositions of mantle minerals in other xenoliths also record transient thermal and chemical anomalies associated with the percolation of slab window-related magmas, which exhibit an “adakite”-type geochemical fingerprint inherited by slab-derived melts produced during ridge subduction and slab window opening event. As these melts percolated through the shallow (7.2–16.8 kbar) and hot (952–1054 °C) lithospheric mantle wedge, they promoted the crystallization of metasomatic clinopyroxene having exceptionally high Li abundances (6–15 ppm). Numerical modeling shows that low degrees (< 10%) of partial melting of this Li-rich and fertile sub-arc lithospheric mantle generates primitive melts having two-fold Li enrichment (~13 ppm) compared with average subduction-zone basalts. Prolonged fractional crystallization of these melts produces extremely Li-enriched silicic rocks, which may stoke the Li inventory of mineralizing fluids in the shallow crust.

## Introduction

Lithium systematics is a powerful geochemical tracer for investigating fundamental processes in the deep Earth, such as crustal recycling in the mantle^1–4^, sedimentary input to subduction zones^5–7^ and slab-to-mantle wedge transfer of fluid-mobile elements^8–11^ that ultimately turn back to the surface via arc lavas^12,13^. Increasing attention in Li geochemistry is also fueled by its key importance to develop electric vehicles technology, which is driving an ever-growing demand of lithium reserves for facing the global transition to a low-carbon economy^14^. Most of lithium supply stems from: (1) granitic pegmatites forming during the late-stage differentiation of highly evolved calc-alkaline magmas^15,16^, (2) basinal brines produced by evaporation of meteoric waters that leach Li from rhyolitic rocks exposed at surface^17,18^, and (3) hydrothermal clays derived by alteration of rhyolitic lavas and volcanic ashes^19^. All these Li ore deposits are closely associated with rhyolitic/granitic source rocks, which crystallized from evolved silicic magmas having extremely high pre-eruptive Li abundances^20,21^. However, the primary factors controlling the Li abundances in magmas ascending through the continental lithosphere, as well as the Li inventory of their mantle source, are still contentious questions^6,22,23^.

The global-scale distribution of Li metallogenic provinces broadly overlaps with the location of convergent margins, such as active continental arcs (e.g., the Andes) and collisional belts (e.g., the Tibetan plateau)^17^, or ancient orogenic hinterlands^16^. The Li over-abundances in arc magmas, coupled with its relative fractionation from elements of similar compatibility in mantle silicates (e.g., Y, Yb)^24^, were originally ascribed to metasomatic Li enrichment in their supra-subduction mantle source^6,22^. Indeed, the lithium inventory of the supra-subduction mantle wedge is thought to be controlled by its extraction from Li-rich subducted lithologies (especially sediments and altered oceanic crust)^5^ via slab-derived hydrous fluids^8,25,26^, which ultimately contribute to the source of arc magmas^6,13,27^. In contrast, compiled global estimates have recently reported that primitive basalts in island arcs have Li abundances (4–8.5 ppm)^23^ and isotopic systematics^28^ that broadly overlap with those of mid-ocean ridge basalts (MORB, ~6.5 ppm Li)^29^. These findings argue against the effective contribution of slab-derived fluids to the Li budget of arc magmas, supporting the idea that Li-enrichment is mostly achieved via prolonged intra-crustal differentiation in thick sectors of continental crust^23^. However, intra-crustal differentiation of average basalts alone^23^ cannot account for the Li contents expected for a magmatic rock to source mineralization^20,21^, nor for the occurrence of several Li-mineralized continental regions where thick crust is lacking (e.g., western United States). Therefore, whether Li-enrichment in magmas is in part inherited by partial melting of a pre-enriched mantle source^6,7^ or is mainly attained by intra-crustal differentiation during the ascent^23^, as well as the mechanisms that may endow Li in the mantle, are still under debate. Resolving this controversy is especially important for targeting mineral camps, as it may provide key tools for predicting the specific geological conditions and environments that predispose the genesis of lithium mineralization in the shallow continental crust^30^.

The Coyhaique mantle xenoliths trapped in Eocene flood basalts from western Patagonia disclose a rare natural window into a sub-arc lithospheric mantle showing chemical and isotopic evidence of subduction-related metasomatism beneath an active continental margin (Chilean Andes, Supplementary Note 1)^31–33^. These mantle samples provide complementary, yet key insights for looking from the mantle source perspective at those mechanisms that govern the lithium inventory in continental arc magmas. In particular, these sub-arc mantle xenoliths allow us to define the primary compositions of subduction-related magmas controlled by the mantle source and partial melting conditions, sorting them out from the possible effects of intra-crustal differentiation and/or crustal contamination.

Here, we report trace element signatures and lithium abundances of minerals in Coyhaique peridotite xenoliths sampling the lithospheric mantle beneath the southern Andes. The data presented in this study suggest that slab dehydration via aqueous fluids was poorly effective on enhancing the Li budget of the supra-subduction mantle wedge. On the other hand, the Li inventory of the sub-arc lithospheric mantle was strongly upgraded by the upwards migration of slab window-related magmas, which bear the contribution of “adakite”-type melts produced by slab melting during ridge-trench collision event. In particular, this metasomatic event boosted the Li contents of the sub-arc mantle peridotites up to those expected for generating Li-rich magmas that may source ore deposition in the overlying crust.

## Results

### Samples and geothermo-oxybarometry

**Figure 1:**
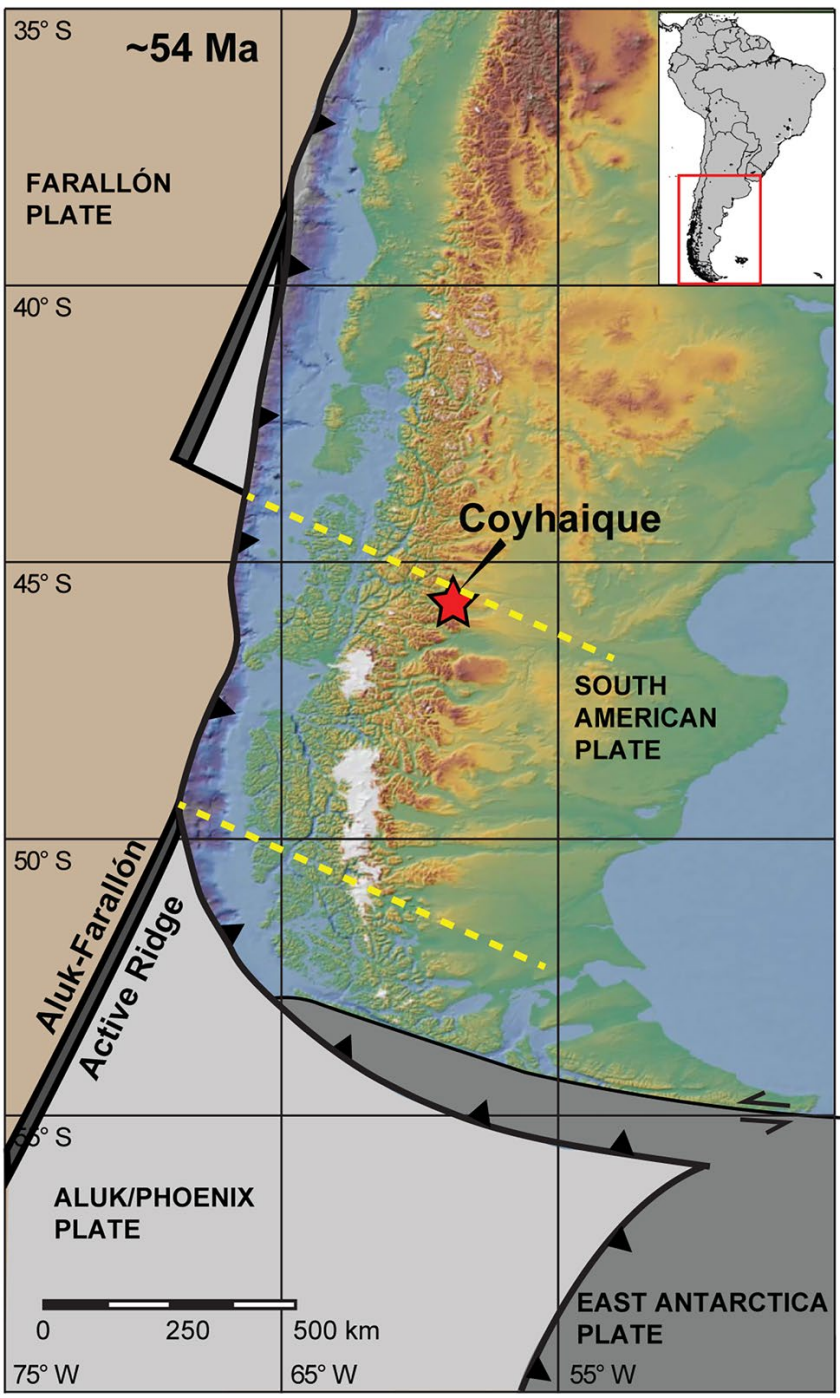
Geotectonic map inferred for Patagonia at ~ 54 Ma showing the reconstructed location of the Balmaceda lava flow bearing the Coyhaique mantle xenoliths (red star), the proposed locations of Farallón (brown), Aluk/Phoenix (light grey), East Antartica (dark grey), and South America plates, and projected slab window (dashed yellow lines) according to ref.^56^ and plate kinematic reconstructions of ref.^97^. Modified after ref.^77^.

The Coyhaique mantle xenoliths are hosted within the Eocene (~54 Ma)^31^ Balmaceda flood basalts (western Patagonia, Fig. 1), which extruded close to the active margin of the Andes subduction zone (~ 320 km east of the Chile trench and ~ 100 km east of the modern volcanic arc, Supplementary Note 1)^31–33^. This magmatic event belongs to the OIB-type plateau mafic magmatism taking place in western Patagonia in response to the opening of an asthenospheric slab window beneath the South America plate^34–36^, which followed the collision of the Aluk-Farallón active ridge against the Chilean trench in the Paleocene^37,38^. The Coyhaique mantle xenoliths selected for this study are anhydrous spinel lherzolites composed of olivine (58–68 vol%), orthopyroxene (17–25 vol%), clinopyroxene (10–15 vol%) and spinel (2–4 vol%) (Supplementary Fig. 1). All samples have rather fertile major element compositions (Al_2_O_3_ = 2.73–3.67 wt%; CaO = 2.39–3.10 wt%; Na_2_O = 0.17–0.25 wt%, Supplementary Table 1 and Supplementary Fig. 2), consistent with previously published data of Coyhaique peridotite xenoliths^31–33^. Rocks have medium to fine-grained (0.5–2 mm) protogranular texture and a weak foliation. Few olivine and orthopyroxene porphyroclasts have undulose extinction and kink bands indicative of mild crystal-plastic deformation. Grain boundaries are straight-lined between olivine-olivine grains, or curvilinear between pyroxene-olivine grains. Subspherical olivine grains are locally included within poikilitic ortho and clinopyroxene (Supplementary Fig. 3). Spinel forms amoeboid crystals with cusps-shaped terminations, commonly intergrown with anhedral orthopyroxene and/or clinopyroxene (Supplementary Fig. 3). Abundant trails of fluid and/or sulfide inclusions are associated with cracks and fractures inside main minerals.

The major element compositions of minerals (Supplementary Table 2) are rather homogeneous at the sample scale and do not show any significant core-to-rim variation, thus attesting to the general chemical equilibration of mineral assemblages. Olivine has Mg# [100 × Mg/(Mg + Fe^2+^)] from 88.9 to 90.1, and NiO and CaO contents < 0.44 wt% and < 0.20 wt%, respectively (Supplementary Fig. 4). Orthopyroxene has Mg# = 89.6–92.2, high Al_2_O_3_ (4.56–5.56 wt%), significant amounts of CaO (0.71–1.00 wt%), and low Cr_2_O_3_ (0.27–0.53 wt%) (Supplementary Fig. 5). Clinopyroxene is rich in Al_2_O_3_ (6.28–7.29 wt%) and Na_2_O (0.89–1.88 wt%), and has Mg# = 88.5–92.8 and CaO = 18.1–19.4 wt% (Supplementary Fig. 6). Spinel has appreciable amounts of TiO_2_ (0.11–0.40 wt%) and displays alumina-rich compositions, which yield low Cr# [Cr^3+^/(Cr^3+^  + Al^3+^  + Fe^3+^) = 0.09–0.15] that are negatively correlated with Mg# in co-existing olivine (Supplementary Fig. 7).

**Figure 2:**
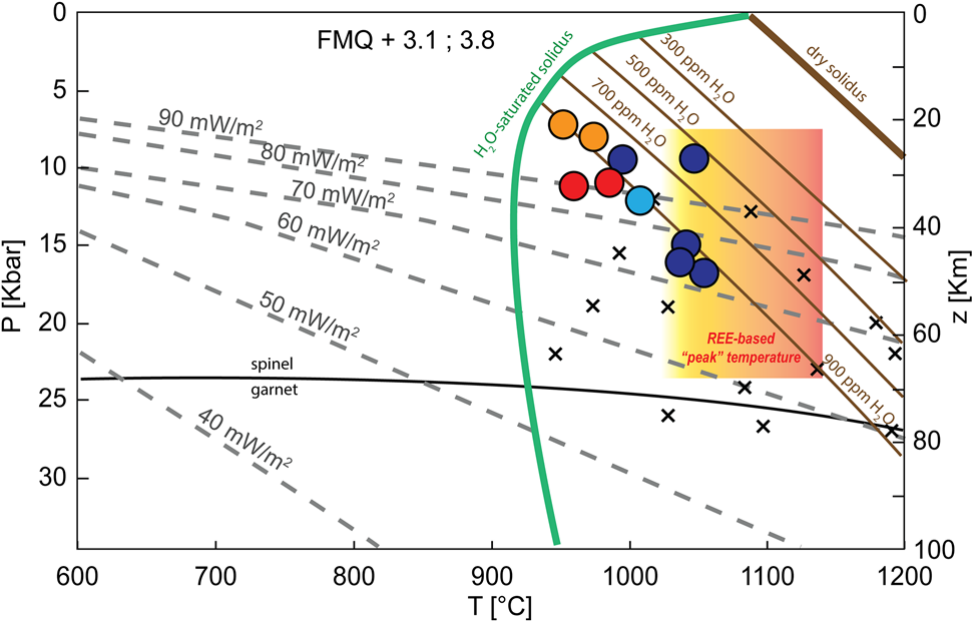
Estimated P–T equilibration conditions of Coyhaique peridotite xenoliths (colored circles), compared to xenoliths from other Patagonian localities (black crosses)^47^. The yellow-to-orange shaded area depicts the range of “peak” temperature conditions experienced by these xenoliths based on the REE-in-two-pyroxene thermometer^50^. The spinel-garnet peridotite transition (black line) is calculated from the spinel composition of the studied samples^45^. The dry solidus (thick brown line), wet solidus with variable H_2_O contents (300–900 ppm, thin brown lines) and water-saturated solidus (green line) for fertile peridotite are from ref.^92^. Model conductive geotherms for different surface heat flow values (dashed grey lines) are from ref.^46^.

The redox equilibration conditions calculated based on the olivine-spinel equilibria^39^ range between + 3.1 and + 3.8 relative to the fayalite-magnetite-quartz buffer (FMQ) (Supplementary Table 3), thus they correspond to the *f*O_2_ values expected for a sub-arc lithospheric mantle pervasively metasomatized via oxidizing slab-derived fluids/melts^40^. We determined the P–T equilibration conditions of the mantle xenoliths by combining the Ca-in-olivine coexisting with clinopyroxene geobarometer^41,42^, with the Ca-in-orthopyroxene geothermometer^43^ and the P-independent Cr-Al-in-orthopyroxene geothermometer^44^. Geothermobarometry calculations were applied to core compositions between adjacent mineral grains with similar Mg# indicating compositional equilibration (Supplementary Table 3). The peridotite xenoliths from Coyhaique yield P–T conditions ranging between 7.2 and 16.8 kbar (± 1.7 kbar) and 952–1054 °C (± 20 °C) (Fig. 2), which are consistent with previous temperature estimates by refs.^32,33^ at an assumed pressure of 15 kbar. The calculated pressure conditions are below the minimum pressure of the spinel-garnet peridotite transition in these xenoliths (24.9–26.9 kbar, Fig. 2), as determined by the spinel composition^45^. The predicted temperature distribution with depth of Coyhaique mantle xenoliths, at the time of their eruption, does not fit any steady-state continental geotherm with constant surface heat flow (Fig. 2)^46^. Similar thermal anomalies usually characterize continental regions with thin crust (< 20 km) and high heat flows produced by ridge subduction and slab window opening events^47–49^, consistent with the Cenozoic geodynamic evolution proposed for western Patagonia^36^. The temperature estimates based on the major elements compositions^43,44^ are 40–120 °C lower than those calculated from REE data on clinopyroxene-orthopyroxene pairs^50^ (Supplementary Fig. 9). Similar discrepancies are generally ascribed to the slower diffusivities of REEs compared to divalent cations in mantle silicates at magmatic temperatures^51–53^. The time-scale modelling for diffusive re-equilibration of REEs (e.g., Yb, see Methods section)^54^ between clino and orthopyroxene reveals that the Coyhaique peridotite xenoliths experienced a high-T heating event (1020–1119 °C, Supplementary material) less than ~4 Ma before their eruption at surface (i.e., ~54 Ma)^31^, followed by their cooling in shallower portions of the lithospheric mantle. The timing of this high-temperature heating event (59–54 Ma) broadly coincides with the onset of slab window opening due to Farallón-Aluk ridge subduction in the Paleocene-Early Eocene^55,56^. Overall, these thermobarometric estimates evidence the development of thermal anomalies associated with the convective thinning of the lithospheric mantle in response to hot asthenosphere upwelling, which was induced by the subduction of the Farallón-Aluk oceanic ridge and the opening of a slab window beneath the South America plate^49^.

### Trace element signatures of minerals

**Figure 3:**
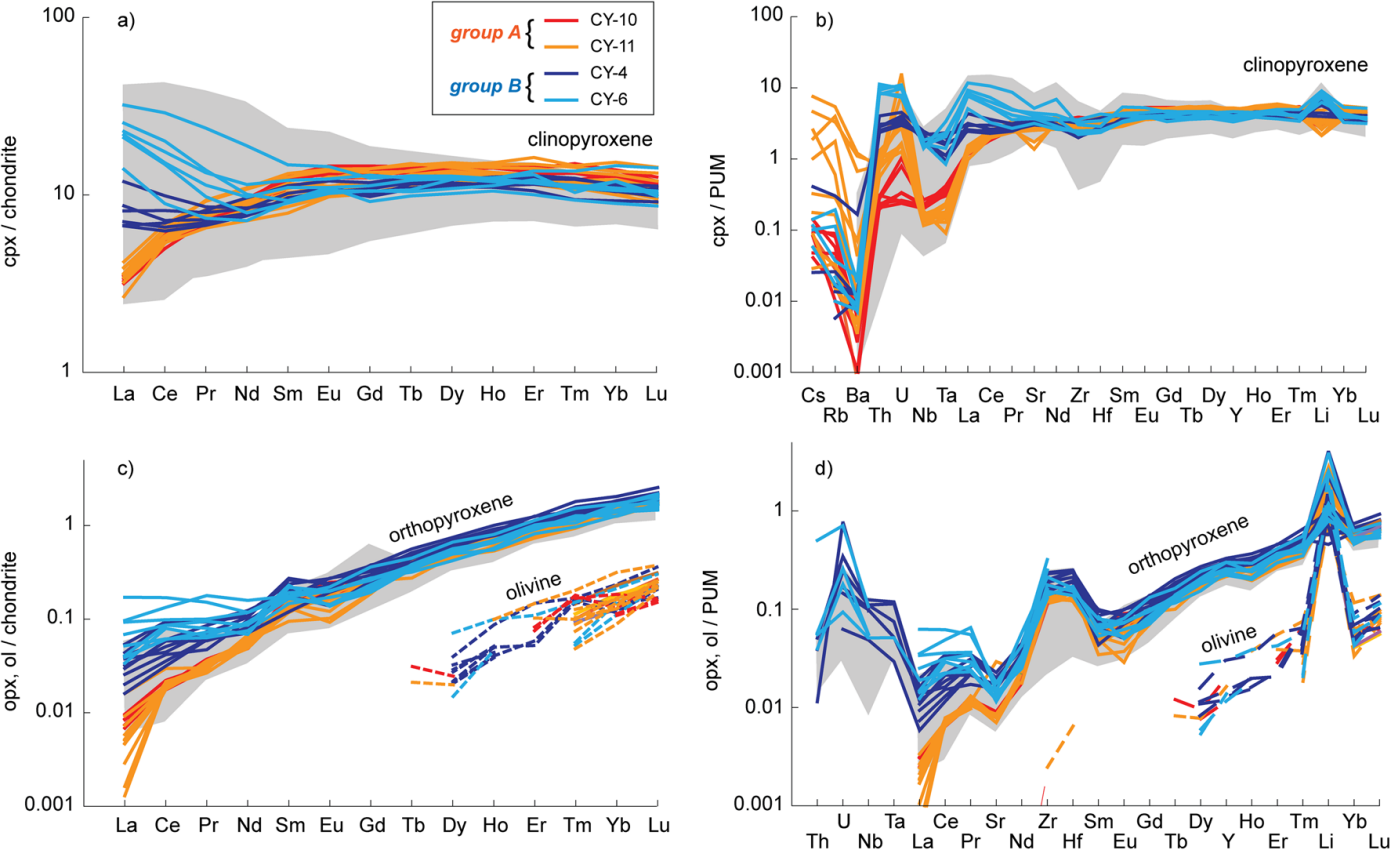
Trace elements systematics of mantle minerals in Coyhaique peridotite xenoliths. Chondrite- and primitive upper mantle (PUM)-normalized concentrations of rare earth elements (REE) and lithophile trace elements, respectively, in clinopyroxene (**a**, **b**), orthopyroxene (solid lines in c, d) and olivine (dashed lines in **c**–**d**) from Coyhaique mantle xenoliths, compared with previously published data from the same locality (grey fields)^33^. Normalizing values from ref.^59^.

Two distinct groups (A, B) of peridotite xenoliths from Coyhaique are recognized based on the contrasting trace element signatures of their constituent pyroxenes (Supplementary Table 4, see also ref.^33^). Clinopyroxene in group A (samples CY-10 and CY-11) has rather homogeneous convex-upward, chondrite-normalized REE patterns with LREE depletion (La_N_/Sm_N_ = 0.26–0.42) and flat MREE-HREE segments at ~ 10 times the chondritic concentrations (Fig. 3a). The PUM-normalized trace element distributions of clinopyroxene in group A xenoliths are characterized by variable enrichments in large ion lithophile elements (LILE: Cs, Rb, Ba) and U, coupled with low concentrations of Nb and Ta, which yield Ba_N_/Nb_N_ = 0.004–8.35 and Rb_N_/Nb_N_ = 0.051–29.33 (Fig. 3b). On the other hand, clinopyroxene in group B xenoliths (samples CY-4 and CY-6) has increasingly positive LREE inflections up to concave-up LREE-enriched patterns (La_N_/Sm_N_ = 0.60–2.73) and MREE-HREE concentrations that are similar to those of clinopyroxene in group A (Fig. 3a). Clinopyroxene in group B generally lacks LILE enrichment, and also has higher Th, Nb and Ta concentrations than in group A, which result in low Ba_N_/Nb_N_ (0.005–0.08) and Rb_N_/Nb_N_ ratios (0.003–0.15; Fig. 3b). The Li concentrations in clinopyroxene from Coyhaique peridotites range between 3.42–7.70 ppm for group A xenoliths (except two outliers with ~12 ppm) and 6.34–15.53 ppm for group B xenoliths (Supplementary Table 4), in agreement with those reported by ref.^33^ in the same locality. These lithium abundances are notably higher than the average values reported for clinopyroxene in moderately depleted to fertile upper mantle (0.4–2.5 ppm Li in clinopyroxene)^57^. In particular, Li over-abundances in group B clinopyroxene yield positive anomalies in the PUM-normalized trace element patterns compared with adjacent elements of similar compatibility (e.g., Yb)^24^ (Fig. 3b). Such positive anomalies are generally absent in group A clinopyroxene (except of the outliers), which rather exhibits slightly negative Li_N_/Yb_N_ ratios.

Orthopyroxene from the two groups of xenoliths also has systematic compositional differences. In all samples, orthopyroxene and olivine have straight-lined, positively-sloping LREE-depleted patterns, with LREE concentrations below detection limits in the case of olivine (Fig. 3c). However, the degree of LREE depletion is much more pronounced in group A orthopyroxene (La_N_/Sm_N_ = 0.01–0.12) than in group B orthopyroxene (La_N_/Sm_N_ = 0.08–0.99), the latter having LREE contents up to 0.1 the chondritic concentrations (Fig. 3c). Moreover, orthopyroxene in group A xenoliths has concentrations of the most incompatible trace elements that are below detection limits, whereas orthopyroxene in group B xenoliths displays much higher abundances of Th, U, Nb, Ta, Sr, Zr and Hf (Fig. 3d). The Li concentrations in orthopyroxene and olivine of Coyhaique peridotite xenoliths vary between 0.73–6.50 ppm and 0.81–3.79 ppm, respectively (Supplementary Table 4), which are higher than those reported in moderately depleted to fertile upper mantle (~0.5–1.3 ppm Li in orthopyroxene, ~1–1.8 ppm Li in olivine)^58^. These lithium overabundances in olivine and orthopyroxene produce strongly positive anomalies in their PUM-normalized trace element patterns (Fig. 3d).

## Discussion

### Metasomatic evolution of the lithospheric mantle wedge beneath western Patagonia

**Figure 4:**
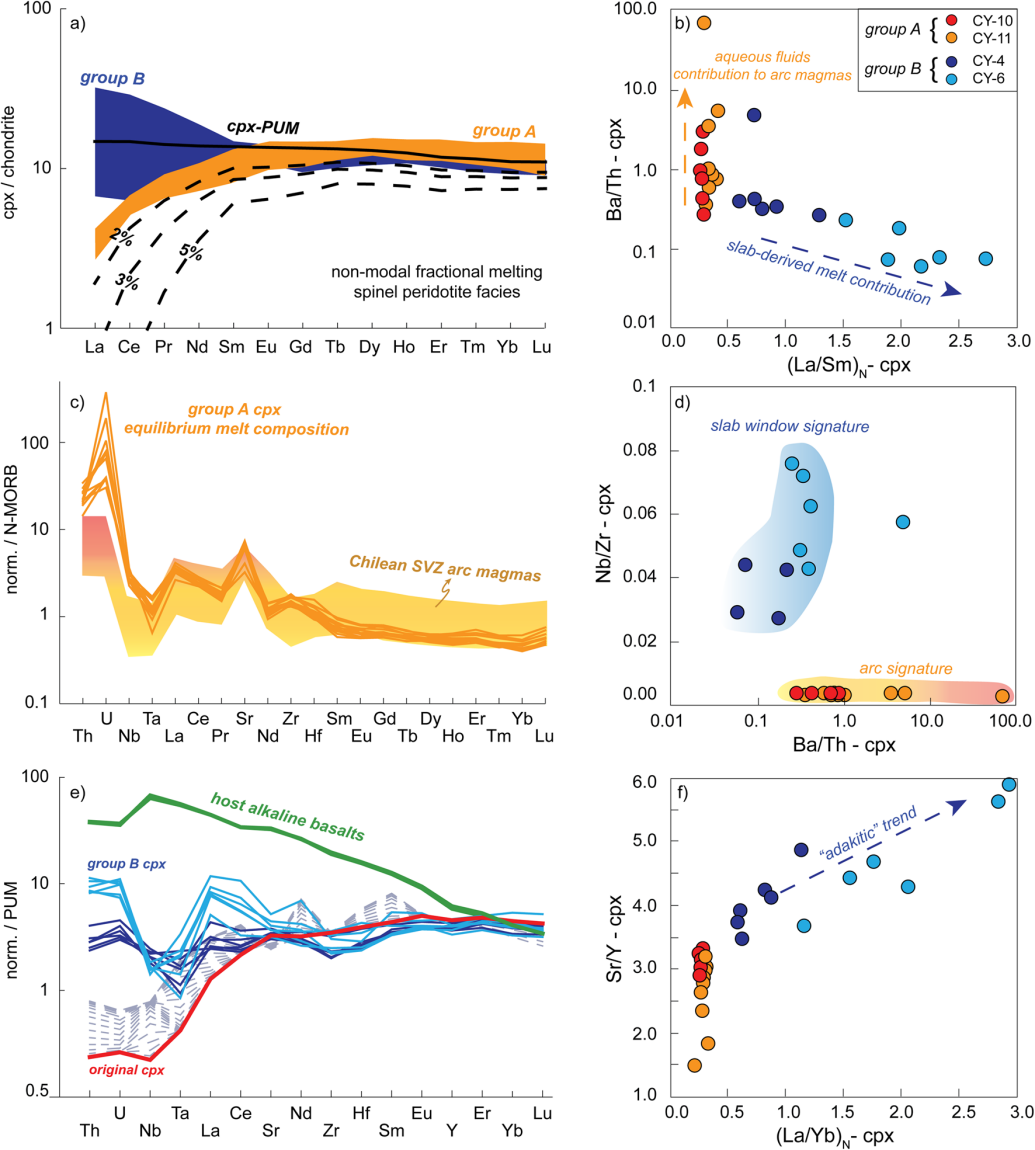
Trace elements signatures and geochemical modelling of clinopyroxene in Coyhaique mantle xenoliths. (**a**) Chondrite-normalized REE patterns of clinopyroxene compared with those produced by non-modal fractional melting (dashed black lines) of clinopyroxene in PUM^59^ in the spinel-peridotite facies (source and melting olivine:orthopyroxene:clinopyroxene modal proportions 0.54:0.28:0.18 and 0.10:0.20:0.68, respectively^96^). (**b**) Correlation between Ba/Th and primitive upper mantle-normalized (La/Sm)_N_ ratios in clinopyroxene from Coyhaique mantle xenoliths. (**c**) N-MORB normalized^29^ trace element patterns of melts in equilibrium with clinopyroxene of Coyhaique mantle xenoliths from group A (orange lines), compared with calc-alkaline volcanic suites of the Chilean Southern Volcanic Zone (shaded orange field)^68,69^. Equilibrium melt compositions were calculated by using the partition coefficients of ref.^98^ for Th, U and Nb, and of ref.^99^ for Ta, Sr, Zr, Hf and REEs. (**d**) Nb/Zr vs. Ba/Th ratios in clinopyroxene from Coyhaique mantle xenoliths. Higher Nb/Zr coupled with low Ba/Th ratios are typical of slab window-related magmas, whereas low Nb/Zr with high Ba/Th are commonly associated with arc magmas from a mantle source fluxed by slab fluids^48^. (**e**) Comparison of trace elements distribution in group B clinopyroxene from Coyhaique (solid blue lines) and those numerically modelled by chromatographic fractionation (dashed grey lines) of the host alkaline basalts (solid green line) interacting with the mantle xenoliths. Chromatographic modelling of host alkaline basalt^31^ percolating for 20 ky with 5 cm/year velocity through 1 km long mantle column was performed following the method presented in ref.^100^. The mantle column was assumed to have a modal composition of depleted mantle and porosity of 1%, and the clinopyroxene starting composition was taken from the most LREE-depleted cpx in the xenolith suite (red line). Partitioning coefficients for all mantle minerals were taken from ref.^95^. (**f**) Sr/Y vs primitive upper mantle-normalized (La/Yb)_N_ ratios in clinopyroxene from Coyhaique mantle xenoliths, showing the “adakitic”-type geochemical trend in clinopyroxene from group B xenoliths.

The studied peridotite xenoliths from Coyhaique have fertile major element compositions relatively enriched in “basaltic” components (i.e., Al_2_O_3_, CaO, Na_2_O), which approach the PUM reservoir (Supplementary Fig. 2)^59^. The Mg# in olivine (88.9–90.1) and Cr# in coexisting spinel (0.09–0.15) shift from the partial melting trend defined by the olivine-spinel mantle array (Supplementary Fig. 7)^60^. These characteristics, together with the presence of poikilitic crystals of ortho and clinopyroxene including olivine grains (Supplementary Fig. 3), support that the Coyhaique peridotite xenoliths experienced metasomatic refertilization by interaction with silicate melts percolating through the lithospheric mantle wedge^61^. In addition, non-modal fractional melting models in the spinel-peridotite field fail to reproduce the REE patterns of clinopyroxene in both groups A and B of xenoliths, even the LREE-depleted compositions of group A under very low melting degrees (< 2%, Fig. 4a). Moreover, HREE abundances in clinopyroxenes approach, or even exceed, those in equilibrium with a PUM-reservoir (Fig. 4a), thus pointing out that silicate melt percolation largely erased the original partial melting signature of these mantle peridotites^31–33^.

**Figure 5:**
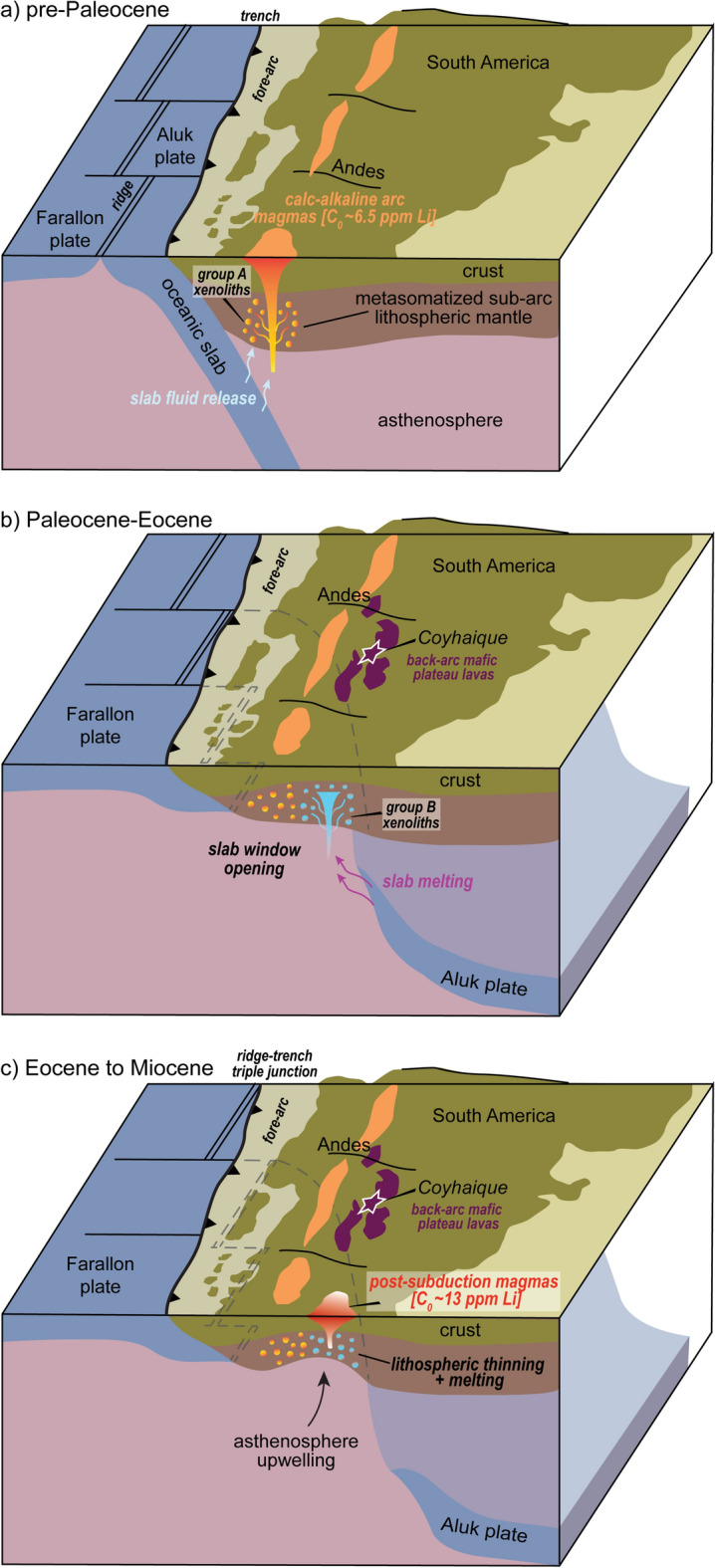
Schematic tectono-magmatic evolution of western Patagonia during the Cenozoic, modified after ref.^36^. Panel (**a**) shows the calc-alkaline arc magmatism taking place along the Chilean active margin before the subduction of the Farallon-Aluk mid-ocean ridge. Panel (**b**) illustrates the tectono-magmatic consequences associated with the Farallon-Aluk ridge collision against the Chilean trench in the Paleocene-Eocene and the opening of an asthenospheric slab window beneath the South America plate. Panel (**c**) shows the tectonic conditions predisposing partial melting events in the metasomatized sub-arc lithospheric mantle of Patagonia, as a consequence of progressive asthenosphere upwelling and convective thinning of the lithosphere associated with slab window opening.

The systematic decoupling between fluid-mobile elements (LILE: Cs, Rb, Ba) and Th-U relative to fluid-immobile elements (HFSE: Nb–Ta) in clinopyroxene from Coyhaique peridotite xenoliths was ascribed by ref.^33^ to a single event of subduction-related mantle metasomatism. However, the different trace element signatures of both clino and orthopyroxene between the two groups of samples (Fig. 3) support that these xenoliths experienced a multi-stage evolution involving the interaction with two different metasomatic agents^62^. The contrasting correlation between Ba/Th and (La/Sm)_N_ ratios recorded by these cpx (Fig. 4b) can be interpreted as due to different contributions of slab-derived aqueous fluids or melts to the source of arc basalts these xenoliths interacted with^63,64^. In fact, while Ba is easily scavenged from the subducted oceanic lithosphere through a wide range of P–T conditions^65,66^, the low solubility of Th in aqueous fluids limits its extraction by slab fluids^65,66^. Therefore, the high Ba/Th ratios of group A clinopyroxenes, coupled with their generally high LILE/HFSE and convex-upward REE patterns (Fig. 3a,b), reflect their interaction with arc magmas having an important contribution in their mantle source of aqueous fluids released by slab-dehydration at sub-arc depths^63,66,67^ (Fig. 5a). This inference is supported by the broad overlap between the compositions of melts in equilibrium with these clinopyroxenes and the calc-alkaline arc lavas of the Chilean Southern Volcanic Zone (Fig. 4c)^68,69^.

On the other hand, clino- and orthopyroxene in group B xenoliths have positively correlated abundances of LREE, Th, Nb and Ta, which points against a major contribution of slab-fluids to the source of metasomatic agents, due to the low solubility of these elements in fluids extracted from dehydrating oceanic lithosphere^65^. The higher Nb/Zr ratios coupled with low Ba/Th in group B cpx (Fig. 4d) recall the geochemical affinity of slab window-related magmas^36,48,70,71^_,_ similar to the OIB-type host alkaline basalts derived by decompression partial melting of anhydrous upwelling asthenosphere^31,34,35^. The variable LREE/HREE fractionation in these cpx (Fig. 3a) could reflect the progressive chromatographic differentiation of such OIB-type alkaline melts as these percolated at decreasing melt fractions through the conductive lithospheric mantle^72,73^. However, numerical modelling of interaction between Coyhaique mantle clinopyroxene and host alkaline basalts experiencing chromatographic fractionation does not reproduce the range of trace elements patterns recorded by group B cpx (Fig. 4e). Moreover, chromatographic differentiation of alkaline basalts during melt percolation cannot explain the depletion of Nb–Ta relative to elements with similar compatibility (e.g., Th-U-La) in these cpx (Fig. 3), which rather suggests a slab imprint in the metasomatizing agent. In particular, decreasing Ba/Th at increasing (La/Sm)_N_ ratios in group B cpx (Fig. 4b) are consistent with the geochemical trend of subduction zones magmas having a slab-derived melt contribution to their mantle source^63^, due to the increasing solubility of Th and LREE in melts produced by slab melting^65^ (Fig. 5b). Furthermore, the positive correlation between Sr/Y vs. (La/Yb)_N_ ratios in group B cpx (Fig. 4f) hints to an “adakite”-type geochemical signature^74^ similar to that recorded by adakite-metasomatized mantle xenoliths from the surrounding Patagonia region^75–77^. In fact, as the ridge-trench collision promoted the opening of a slab window in the Paleocene-Eocene, partial melting at thinned slab edges may have generated “adakite”-type melts^48,78^, which are expected to imprint the geochemical signature of the slab window magmas produced in the surrounding asthenosphere^71^ (Fig. 5b).

Therefore, the trace element signatures of clino- and orthopyroxene in the Coyhaique peridotite xenoliths record a multi-stage metasomatic evolution involving the interaction with: (1) subduction-related arc magmas enriched in fluid-mobile elements added by slab-derived aqueous fluids, consistent with the calc-alkaline volcanism in the active margin of the Chilean Andes since the Mesozoic^69^ (Fig. 5a), and (2) slab window-related magmas bearing the source contribution of slab-derived melts with “adakite”-type geochemical affinity^48,78^ (Fig. 5b).

### Lithium enrichment in convergent margins

**Figure 6:**
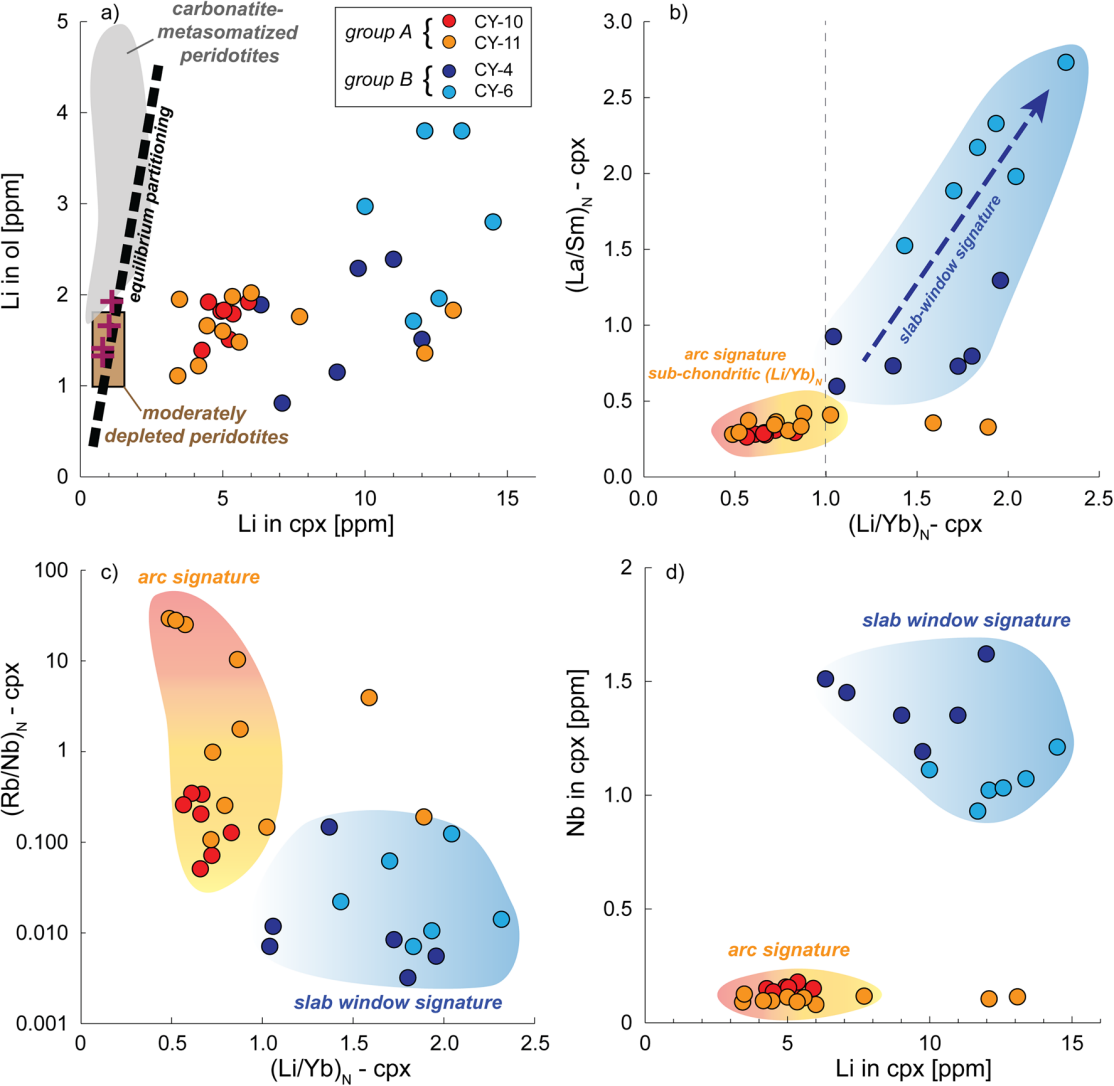
Geochemical implications of the metasomatic evolution of Coyhaique peridotite xenoliths for the Li inventory of the sub-arc lithospheric mantle. (**a**) Lithium concentrations (ppm) in coexisting olivine and clinopyroxene in Coyhaique peridotite xenoliths, compared with equilibrium partitioning^58^ and corresponding values in depleted continental peridotites (purple crosses)^83^. The brown and grey areas indicate the compositional ranges in moderately depleted peridotites and carbonatite-metasomatized peridotites, respectively^57^. Covariations of (Li/Yb)_N_ with (La/Sm)_N_ (**b**) and (Rb/Nb)_N_ (**c**), and of absolute abundances of Li with Nb (ppm) (d) in clinopyroxene grains in Coyhaique peridotite xenoliths.

The distribution of Li between coexisting clinopyroxene (3.42–14.54 ppm) and olivine (0.81–3.80 ppm) in Coyhaique peridotite xenoliths diverge from the equilibrium partitioning trend based on inter-mineral partition coefficients (Fig. 6a)^58^, documenting disequilibrium enrichment processes associated with mantle metasomatism^57,79–82^. Reaction with silicate melts thus enhanced the Li inventory of the supra-subduction mantle wedge beneath western Patagonia^33^ by crystallizing metasomatic clinopyroxene with Li contents that are far beyond the average values in moderately depleted to fertile peridotites^57,58,83–85^. The positive correlation between Li abundances and slow-diffusive elements (e.g., La), as expressed by (Li/Yb)_N_ vs. (La/Sm)_N_ co-variation in clinopyroxenes (Fig. 6b), supports that Li-enrichment was not controlled by diffusive re-equilibration with host lavas^86^, but was primarily linked to the metasomatic evolution of the peridotite xenoliths before their eruption at surface.

The Li-enrichment of the mantle wedge is generally ascribed to a dominant contribution of subducting sediments^6^. For instance, subducting sediments beneath the Chile trench have high Li contents (40–60 ppm,)^87^, on average with Li sedimentary input in subduction zones^5^, thus potentially providing significant Li budget to the otherwise depleted upper mantle wedge. The minor Li enrichment in group A clinopyroxenes (median 5.13 ppm Li; Figs. 3 and 6a), coupled with their sub-chondritic (Li/Yb)_N_ ratios (except of two outliers; Fig. 6b), support that slab dehydration at sub-arc depths was poorly effective on enhancing the Li budget of the arc magmas that interacted with these xenoliths^1,79,86^. This observation may in part reflect the rapid removal of Li from slab-derived fluids due to diffusive re-equilibration with mantle wedge peridotites^28,80^. Moreover, the lack of correlation between Li contents and proxies of slab-fluids contribution (e.g., Ba, Rb, Fig. 6c) is consistent with the low solubility of Li in aqueous fluids released by slab dehydration^65^. These observations further support that a relevant fraction of the subducted lithium inventory is retained in the oceanic lithosphere during prograde dehydration^1,8,9^, being possibly cycled back at greater depths in the convective mantle^1^.

On the other hand, remarkably high Li abundances are reported for clinopyroxene in group B xenoliths (median 11.4 ppm Li, Fig. 6a), which interacted with slab window-related magmas having an “adakite”-type geochemical signature. In these clinopyroxenes, the Li enrichment is coupled with higher abundances of fluid-immobile and highly incompatible elements (e.g., Nb, Fig. 6d), which may be significantly remobilized in slab melts^65^. These observations thus suggest that melting of dehydrated oceanic lithosphere (e.g., eclogitic metasediments and/or oceanic crust)^65^, possibly triggered by local thermal anomalies arising during ridge-trench collision and slab window opening^48^, is the main geological process that may drive Li-enrichment in continental arcs. Notably, Li over-abundances in mantle clinopyroxene comparable to those reported here have been ascribed to the interaction with EM1-like metasomatic agents with the imprint of deeply recycled, dehydrated oceanic lithosphere^88,89^.

**Figure 7:**
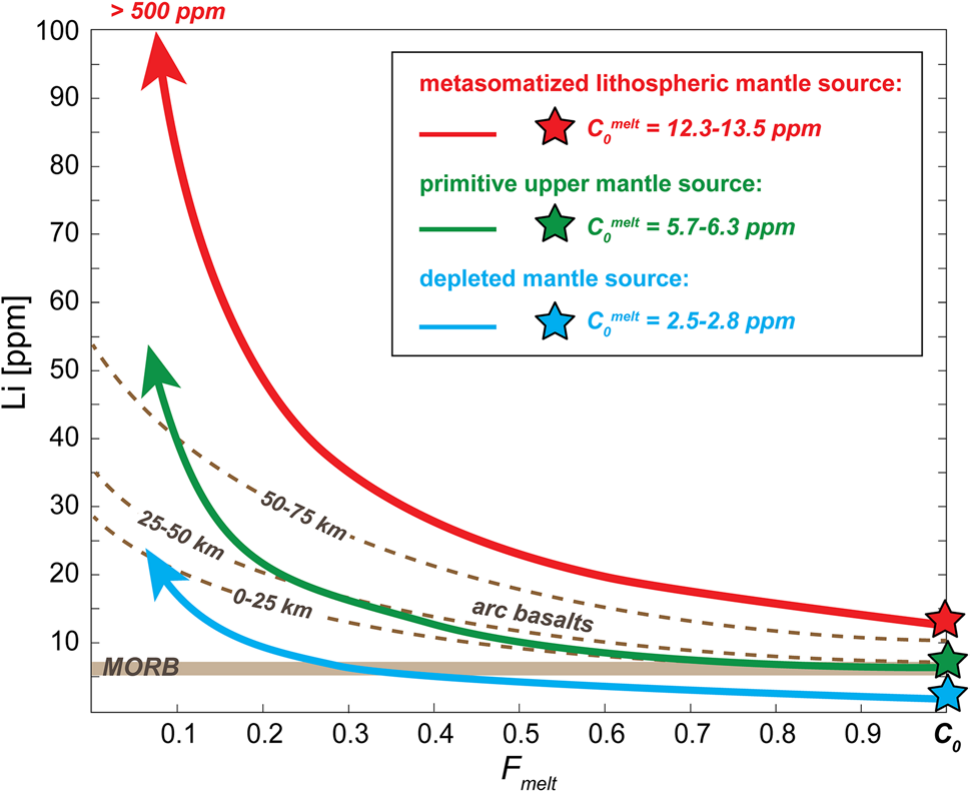
Evolution of Li concentrations by fractional crystallization in mantle-derived magmas generated by distinct mantle sources. Initial lithium concentrations in primitive melts (*C*_*0*_^*melt*^) were calculated by low degrees (*F* < 0.10) of non-modal fractional melting of group B Coyhaique peridotites (red star), primitive upper mantle source (PUM, green star)^59^ and depleted mantle (blue star)^90^. Average lithium abundances in MORB^29^ and Li evolution trends in arc lavas for variable crustal thickness (25 km, 50 km and 75 km)^23^ are shown for comparison. The parameters used in the calculations are listed in the Supplementary Table 5.

Mass balance calculations using Li median concentrations of rock-forming minerals (olivine, orthopyroxene and clinopyroxene) and their modal proportions in group B peridotite xenoliths from Coyhaique yield whole-rock Li abundances ranging between 2.67–3.59 ppm, which are twice-to-five times higher than average Li contents of conventional upper mantle reservoirs (e.g., 0.70 ppm in depleted mantle^90^; 1.6 ppm in primitive upper mantle^59^; 1.6–1.8 ppm in MORB mantle source^83^). The Li concentrations calculated for Coyhaique peridotite xenoliths are also remarkably higher than those previously documented for subduction-metasomatized peridotites from continental arc settings (0.9–1.6 ppm Li)^86,91^. Notably, the P–T conditions inferred for this set of mantle xenoliths are beyond the wet solidus of mantle peridotites (Fig. 2)^92^, thus supporting the hypothesis that the fertile and Li-rich lithospheric mantle beneath western Patagonia may experience low degrees of melting upon its progressive convective thinning associated with the slab window opening event^48^ (Fig. 5c). Under the P–T-*f*O_2_ conditions recorded by the Coyhaique xenoliths (Supplementary Table 5), thermodynamic calculations using the pMELTS software^93^ predict the generation of basaltic magmas by 2–10% partial melting of hydrated mantle (0.3–0.7 wt% H_2_O) at 10–15 kbar and 1000–1200 °C (Supplementary Table 5). We then calculated the Li abundances in primary magmas by non-modal fractional melting Eq. ^94^:

$${C}_{melt}^{Li}={ \frac{{C}_{peridotite}^{Li}}{F}*[1-(1-\frac{P*F}{D}) }^{(\frac{1}{D})}]$$

which expresses the composition of an aggregate melt (*C*^*Li*^_*melt*_) formed by the integration of infinitesimal melt fractions continuously separated from the peridotite residuum, as a function of the maximum bulk Li concentration of Coyhaique peridotite xenoliths (*C*^*Li*^_*peridotite*_ = 3.59 ppm), the bulk partition coefficient of Li (*D*^*Li*^ = 0.258) experimentally determined for lherzolite by ref. ^95^, the mineral proportions entering the melt at 15 kbar (*p*_*ol*_*:p*_*opx*_*:p*_*cpx*_ = 0.10:0.20:0.68)^96^, and over the range of melting degrees (*F* < 10%) predicted by pMELTS (Supplementary Table 5). The inferred Li concentrations in primary magmas sourced by the metasomatized lithospheric mantle beneath Coyhaique (12.5–13.5 ppm Li, red star in Fig. 7) are twice times higher than those globally reported for asthenosphere-derived magmas, such as MORBs (6.5 ppm Li)^29^ and primitive arc basalts (4–8 ppm Li)^23^ (Figs. 5, 7). Equivalent low degrees of partial melting (*F* = 2–8%) for an average primitive upper mantle source (PUM)^59^ or depleted mantle (DM)^90^ do not produce any significant Li enrichment in primary magmas (5.5–6.5 ppm Li, Supplementary Table 5), consistent with the moderately incompatible behavior of Li during mantle melting^24,95^. These calculations show that the high Li abundances in primitive magmas rising through thick continental arcs cannot be ascribed only to low degrees of melting of an “average” mantle source^23^. In particular, melting extent suppression in the mantle wedge via crustal thickening^23^ alone is not sufficient for upgrading the Li contents in mantle-derived magmas towards those expected to source ore mineralization in the overlying crust. Rather, these findings support that the Li over-abundances in certain magmatic suites require Li pre-enrichment in their mantle source^6,22^, mainly mediated by metasomatic addition via slab-derived melts instead of aqueous fluids (Fig. 5).

Then, we estimated the Li abundances in magmas undergoing differentiation while ascending through the continental lithosphere (Fig. 6) by employing the fractional crystallization equation:

$${C}_{residual melt}^{Li}={C}_{0}^{Li}*{{F}_{melt}}^{(D-1)}$$

where *C*_*0*_ is the initial lithium concentration in primary melt, *F*_*melt*_ the fraction of residual melt left after crystallization, and *D* the bulk partition coefficient of Li in arc volcanic rocks of basaltic-andesitic composition (*D* = 0.2)^23,24^. Evolved magmas sourced by a metasomatized lithospheric mantle similar to group B xenoliths exhibit 2–3 fold enrichment in Li relative to average basalts sourced by MORB mantle (Fig. 7)^70^ and arc basalts differentiated in continental crust with different thickness (Fig. 7)^23^. Notably, the last batches of melts issued from a Li-rich source like the Coyhaique lithospheric mantle attain exceptionally high Li abundances (> 500 ppm) similar to those expected for a magmatic rock that feeds ore mineralization via alteration/weathering and concentration in evaporative brines^20,21^.

All these findings support that the genesis of ore-productive Li-rich magmas in convergent margins requires a pre-enriched source region in the metasomatized sub-arc lithospheric mantle (Fig. 5). The pre-enrichment of lithium in the supra-subduction mantle is mainly achieved by interaction with slab window-related magmas, which have an important contribution of slab-derived melts in their mantle source. The minor Li enrichment caused by metasomatic interaction with arc magmas with a major imprint of slab-derived fluids further corroborates the low effectiveness of these fluids on remobilizing Li in the supra-subduction mantle wedge. Indeed, the data presented in this study support that a large fraction of the subducted Li inventory persists in the dehydrated oceanic lithosphere, being possibly recycled in the deep convective mantle in case the slab does not melt. Partial melting of a lithospheric mantle metasomatized via slab-derived melts, coupled with extreme intra-crustal differentiation of melts by fractional crystallization, may properly account for the extreme Li enrichment of the igneous rocks that source ores deposits under favorable conditions of alteration and weathering in the shallow crust.

## Methods

### Whole-rock compositions

The whole-rock major element compositions of peridotite xenoliths from Coyhaique were determined by X-ray fluorescence (XRF) on representative aliquots of pulverized sample, by using a ThermoARL Advant’XP + sequential XRF spectrometer at the GeoAnalytical Lab of the Washington State University (USA). Sample preparation was performed by single-low dilution fusion technique. Pulverized samples were prepared by grinding in a swing mill with tungsten carbide surface and weighing with di-lithium tetraborate flux. The mixed powders were then emptied into graphite crucibles and loaded into a muffle furnace at 1000 °C. After cooling, the bead was reground, refused and polished on diamond laps, and then loaded into the XRF spectrometer. A rhodium target was run at 50 kV and 50 mA with full vacuum and a 25 mm mask for all analyses. The concentrations of elements in the unknown samples were measured by comparing the X-ray intensity for each element with the intensity of USGS standard samples (AGV-2, BCR-2 and GSP-2). The intensities for all elements were corrected automatically for line interference and absorption effects due to all the other elements using the fundamental parameter method.

### Major and trace elements mineral data

The major element compositions of silicate minerals (expressed in wt.% of SiO_2_, Al_2_O_3_, TiO_2_, Cr_2_O_3_, MgO, Na_2_O, MnO, FeO, NiO, CaO and K_2_O) were determined by using a JEOL JXA-8230 electron microprobe analyzer (EMPA) at the Serveis Cientificotècnics of Universitat de Barcelona (Spain). The analyses were conducted by employing 20 kV accelerating voltage and 15–20 nA beam current for a 5 μm spot-size beam and 6.5 nA beam current. Counting time for each element was 20 s for peak and 10 s for background, and ZAF correction was applied online. Calibration was performed using natural and synthetic standards. The major element composition of spinel (expressed in wt% MgO, Al_2_O_3_, SiO_2_, FeO, MnO, TiO_2_, NiO, ZnO, CoO, CaO, Cr_2_O_3_ and V_2_O_5_) was determined by using a CAMECA SX 100 EMP equipped with five wavelength-dispersive spectrometers (WDS) bearing LPET, LLIF and LTAP crystals at the Centro de Instrumentación Científica of the Universidad de Granada (Spain). Analytical conditions were 20 kV accelerating voltage and 20 nA beam current for a 5 μm spot-size beam. Counting times for Mg, Ti, Ca, Cr and V were 50 s for peak and 25 s for background; for Al and Si were 40 s for peak and 20 s for background; for Fe, Mn, Ni, Zn and Co were 30 s for peak and 15 s for background.

The trace element compositions of clinopyroxene, orthopyroxene and olivine were determined by laser ablation inductively coupled plasma mass spectrometry (LA-ICP-MS) on polished thin sections, by coupling an Agilent 8800 QQQ ICP-MS with a Photon Machine Analyte G2 excimer 193 nm laser at the Instituto Andaluz de Ciencias de la Tierra (CSIC-Universidad de Granada, Spain). The thin sections were put into a double volume ablation cell, and supplied with a He gas flow of 9.7 1/min. Maximum sensibility of analysis was achieved by employing an energy density of 8 J/cm^2^ for a 110 μm spot-size and 30 Hz repetition rate. The ablation mode was static spot and performed as automatic driven positioning. Integration time for all analyses was 60 ms for each mass and the validation standard BHVO-2G (synthetic glass, IAG-USGS) was included between the unknowns to check the accuracy and precision of the analysis. Data reduction was performed using Iolite 2.5 software package (Paton et al., 2011) on the IgorPro platform by the careful inspection of time-resolved spectra to check for the stability and homogeneity of signals during ablation time. Elements were calibrated using the synthetic glass NIST611 SRM.

### Geothermometry calculations based on pyroxenes REE data

The “peak” temperature conditions attained by Coyhaique mantle xenoliths were determined by employing the REE-in-two-pyroxene method of ref.^50^ on the same mineral pairs previously used for major elements-based thermometers. Temperature inversion was performed by extrapolating best-fit lines on ln(*D*)-*A* vs. *B*/1000 diagrams, where *A* is a function of major elements composition of the clino- and orthopyroxene and *B* a function of ionic radii of the REEs (Supplementary Figure 8). Quality check of data consistency was assessed by plotting the REE partitioning coefficients between orthopyroxene and clinopyroxene (*D*^opx/cpx^) as a function of the REEs ionic radii. The time scales expected for diffusive re-equilibration of REEs between orthopyroxene and clinopyroxene was calculated following the method presented in ref.^54^, based on the pyroxenes grain size (2 mm for opx, 1 mm for cpx) and the diffusion coefficients of Yb at the temperature conditions at which the studied xenoliths lastly equilibrated in the mantle^51,52^.

### Supplementary Information

Supplementary Information 1.

Supplementary Table 1.

Supplementary Table 2.

Supplementary Table 3.

Supplementary Table 4.

Supplementary Table 5.

## Data Availability

The data generated in this study are available in the Supplementary file of the article.
