# Supplementary material for: Slab melting boosts the mantle wedge contribution to Li-rich magmas
Source: Sci Rep. 2024 Jul 2;14:15168. doi: 10.1038/s41598-024-66174-y (PMC11219803; doi:10.1038/s41598-024-66174-y)
Supplement: Supplementary file 1 — Supplementary Information 1. [file 41598_2024_66174_MOESM1_ESM.pdf]

Supplementary Information to:

"Slab melting boosts the mantle wedge contribution to Li-rich magmas"

Erwin Schettino[1,2,3], Igór González-Pérez[3], Claudio Marchesi[2,3], José María González-Jiménez[2], Michel Grégoire[4], Romain Tilhac[2], Fernando Gervilla[2,3], Idael F. Blanco-Quintero[5], Alexandre Corgne[6], and Manuel E. Schilling[6]

[1]Department of Earth Sciences, ETH Zürich, Clausiusstrasse 25, 8092 Zürich, Switzerland;

[2]Instituto Andaluz de Ciencias de la Tierra (IACT), Consejo Superior de Investigaciones Científicas-Universidad de Granada, Avenida de las Palmeras 4, 18100 Armilla Spain;

[3]Departamento de Mineralogía y Petrología, Universidad de Granada, Avenida Fuentenueva s/n, 18002 Granada, Spain; [4]Géosciences Environnement Toulouse (GET), Observatoire Midi Pyrénées, CNRS-CNES-IRD-Université Toulouse III, 14 Av. E. Belin, 31400 Toulouse, France;

[5]Departamento de Ciencias de la Tierra y del Medio Ambiente, Facultad de Ciencias, Universidad de Alicante,03690 Alicante, Spain; [6]Instituto de Ciencias de la Tierra, Facultad de Ciencias, Universidad Austral de Chile, Valdivia 5090000, Chile.

**corresponding author: eschettino@ethz.ch*

Supplementary Note 1 – Geological Setting. The active margin of western Patagonia (southern Andes) represents a long-lived subduction system, being active since the Late Jurassic, which experienced two episodes of ridge collision during the Cenozoic[1]. The present-day configuration of the region is characterized by the continuous subduction (since Miocene) of the Nazca-Antarctic spreading ridge beneath the South American plate[2], forming the trench-ridge-trench Chilean triple junction (Fig. 1)[3]. An older Paleocene event of ridge subduction was caused by the collision of the Farallon-Aluk active ridge against the Chilean trench, which drove the opening of an asthenospheric slab-window beneath the South America plate[4,5]. The Paleocene slab window

opening caused a hiatus in the calc-alkaline magmatism along the arc axis of southern Andes, and a shift towards a voluminous mafic plateau volcanism in the back-arc region during the Cenozoic[1]. This tectono-magmatic cycle involved the extrusion of alkaline flood basalts having OIB-like geochemical signatures attributed to a “hot” asthenospheric mantle source[6,7], which locally brought to the surface peridotite mantle xenoliths sampling the supra-subduction lithospheric mantle[8]. The peridotite mantle xenoliths selected for this study come from the Coyhaique lava flow (Fig. 1), which belongs to the Eocene (59-44 Ma) Balmaceda flood basalts extruding in the Patagonian back-arc region[6,9,10]. These mantle xenoliths are located ~ 320 km east of the Chile trench and ~ 100 km east of the modern volcanic arc, thus they sample among the portions of SCLM closest to an active margin of subduction zone[10,11].

Supplementary references

1. Aragón, E., Pinotti, L., Fernando, D., Castro, A., Rabbia, O., Coniglio, J., Demartis, M., Hernando, I., Cavarozzi, C. E., & Aguilera, Y. E. (2013). The Farallon-Aluk ridge collision with South America: Implications for the geochemical changes of slab window magmas from fore-to back-arc. *Geoscience Frontiers, 4*(4), 377-388.
2. Sanhueza, J., Yáñez, G., Buck, W. R., Araya Vargas, J., & Veloso, E. (2023). Ridge subduction: Unraveling the consequences linked to a slab window development beneath South America at the Chile Triple Junction. *Geochemistry, Geophysics, Geosystems, 24*(9), e2023GC010977.
3. Cande, S. C., & Leslie, R. B. (1986). Late Cenozoic tectonics of the southern Chile trench. *Journal of Geophysical Research: Solid Earth, 91*(B1), 471-496.

4. Forsythe, R., & Nelson, E. (1985). Geological manifestations of ridge collision: Evidence from the Golfo de Penas-Taitao Basin, southern Chile. *Tectonics*, *4*(5), 477-495.
5. Thorkelson, D. J. (1996). Subduction of diverging plates and the principles of slab window formation. *Tectonophysics*, *255*(1-2), 47-63.
6. Ramos, V. A., & Kay, S. M. (1992). Southern Patagonian plateau basalts and deformation: backarc testimony of ridge collisions. *Tectonophysics*, *205*(1-3), 261-282.
7. Kay, S. M., Gorring, M., & Ramos, V. A. (2004). Magmatic sources, setting and causes of Eocene to Recent Patagonian plateau magmatism (36 S to 52 S latitude). *Revista de la Asociación geológica Argentina*, *59*(4), 556-568.
8. Schilling, M. E., Carlson, R. W., Tassara, A., Conceição, R. V., Bertotto, G. W., Vásquez, M., Muñoz, D., Jalowitzki, T., Gervasoni, F., & Morata, D. (2017). The origin of Patagonia revealed by Re-Os systematics of mantle xenoliths. *Precambrian Research*, *294*, 15-32.
9. Demant, A., Hervé, F., Pankhurst, R., & Suárez, M. (1996). Geochemistry of Early Tertiary back-arc basalts from Aysén, southern Chile (44-46 S.): geodynamic implications. *Third ISAG, St Malo*, 17-19.
10. Jalowitzki, T., Gervasoni, F., Conceição, R. V., Orihashi, Y., Bertotto, G. W., Sumino, H., Schilling, M. E., Nagao, K., Morata, D., & Sylvester, P. (2017). Slab-derived components in the subcontinental lithospheric mantle beneath Chilean Patagonia: Geochemistry and Sr–Nd–Pb isotopes of mantle xenoliths and host basalt. *Lithos*, *292*, 179-197.
11. Novais-Rodrigues, E., Jalowitzki, T., Gervasoni, F., Sumino, H., Bussweiler, Y., Klemme, S., Berndt, J., Conceiçao, Schilling, M. E., Bertotto, G. W., & Teles, L. (2021). Partial melting and subduction-related metasomatism recorded by geochemical and

isotope (He-Ne-Ar-Sr-Nd) compositions of spinel lherzolite xenoliths from Coyhaique, Chilean Patagonia. *Gondwana Research, 98*, 257-276.

12. Streckeisen, A. (1974). Classification and nomenclature of plutonic rocks recommendations of the IUGS subcommission on the systematics of igneous rocks. *Geologische Rundschau*, *63*, 773-786.
13. Herrmann, W., & Berry, R. F. (2002). MINSQ–a least squares spreadsheet method for calculating mineral proportions from whole rock major element analyses. *Geochemistry: Exploration, Environment, Analysis*, *2*(4), 361-368.
14. McDonough, W. F. & Sun, S. S (1995). The composition of the Earth. *Chemical Geology* **120**, 223-253.
15. Arai, S. (1994). Characterization of spinel peridotites by olivine-spinel compositional relationships: review and interpretation. *Chemical geology*, *113*(3-4), 191-204.
16. Pearce, J. A., Barker, P. F., Edwards, S. J., Parkinson, I. J., & Leat, P. T. (2000). Geochemistry and tectonic significance of peridotites from the South Sandwich arc-basin system, South Atlantic. *Contribution to Mineralogy and Petrology*, 139, 36-53.
17. Liang, Y., Sun, C., & Yao, L. (2013). A REE-in-two-pyroxene thermometer for mafic and ultramafic rocks. *Geochimica et Cosmochimica Acta*, 102, 246-260.
18. Brey, G. P. & Köhler, T. (1990). Geothermobarometry in four-phase lherzolites II. New thermobarometers, and practical assessment of existing thermobarometers. *Journal of Petrology, 31*, 1353-1378.

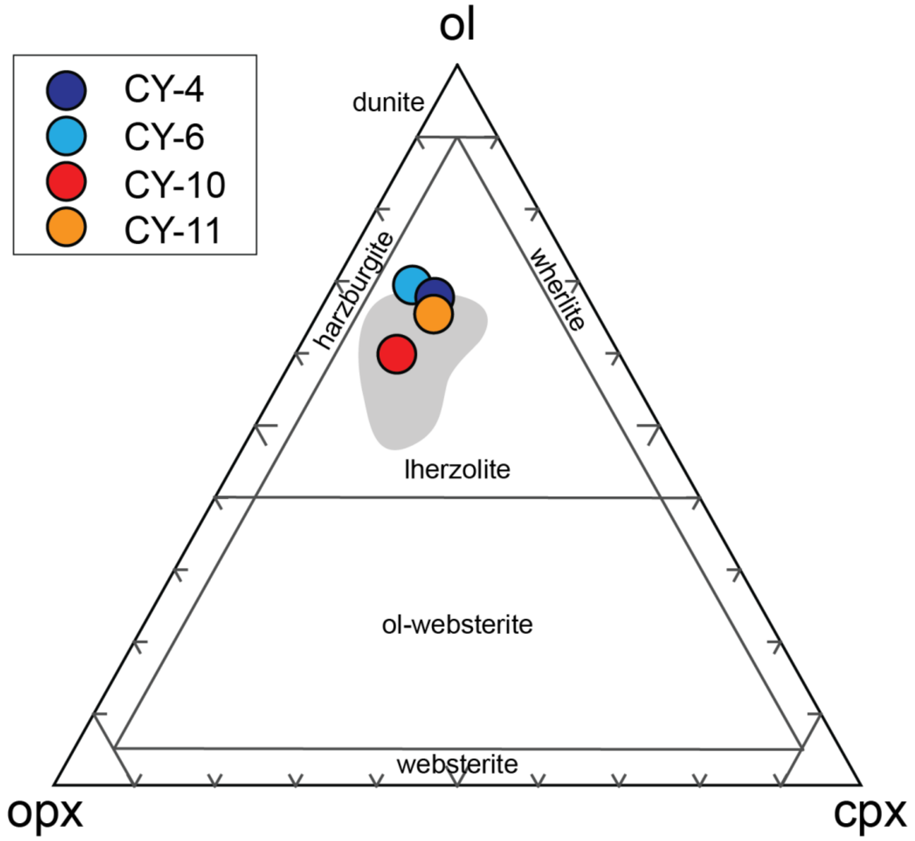

Figure S1. Modal proportions (vol. %) of the Coyhaique mantle xenoliths (circles) plotted on the ternary classification diagram of ref. [12], compared with previously published data of xenoliths from the same locality (grey field, ref. [11]). Modal proportions were estimated by mass balancing whole-rock compositions with mineral chemical data for all phases present in the xenoliths (ref. [13]).

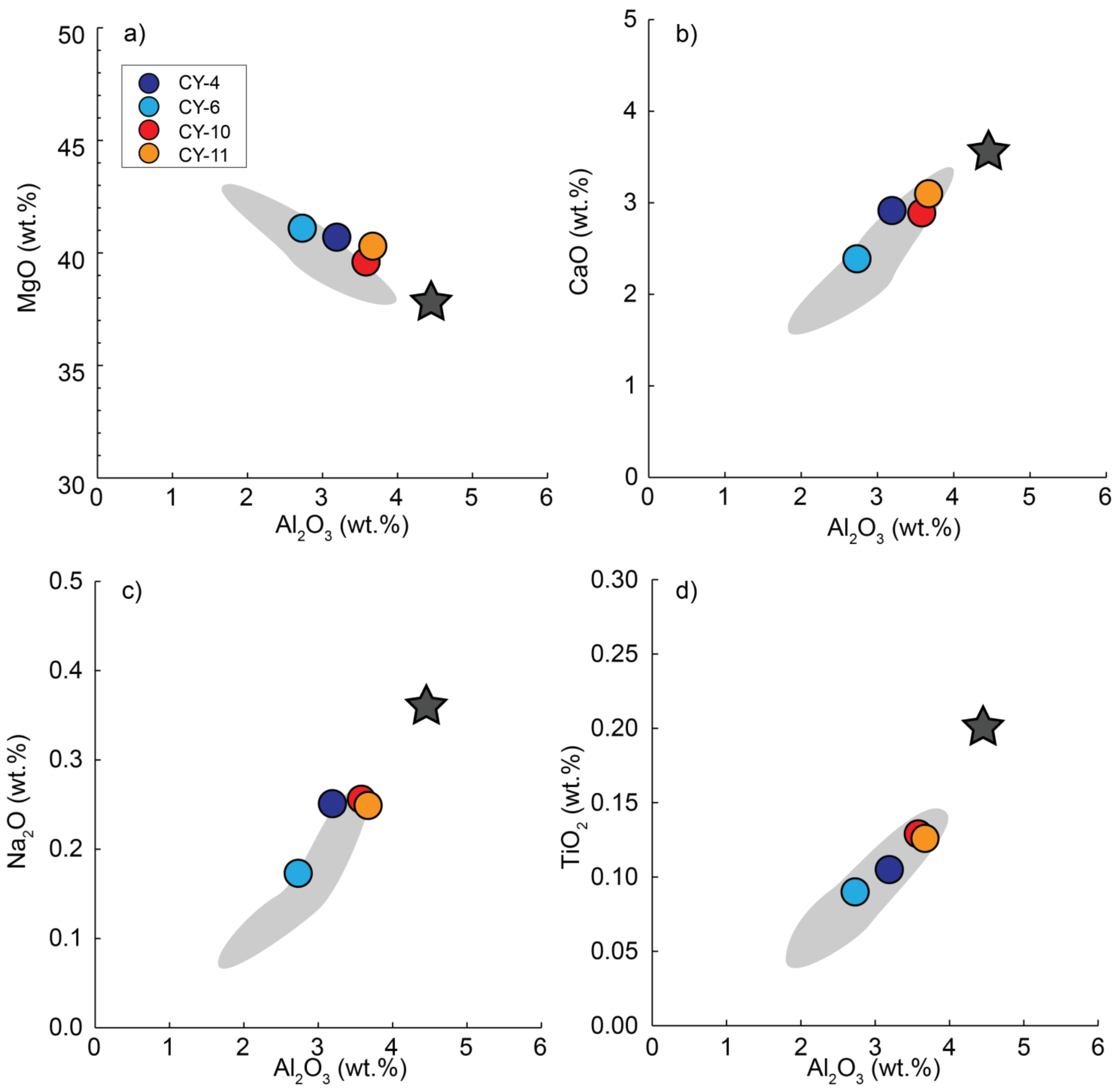

Figure S2. Co-variation diagrams of whole-rock Al_2O_3 versus MgO (a), CaO (b), Na_2O (c) and TiO_2 (d) abundances (wt.%) of Coyhaique peridotite xenoliths, compared with primitive upper mantle (PUM – grey star)[14] and previously published data from the same locality (grey fields)[11].

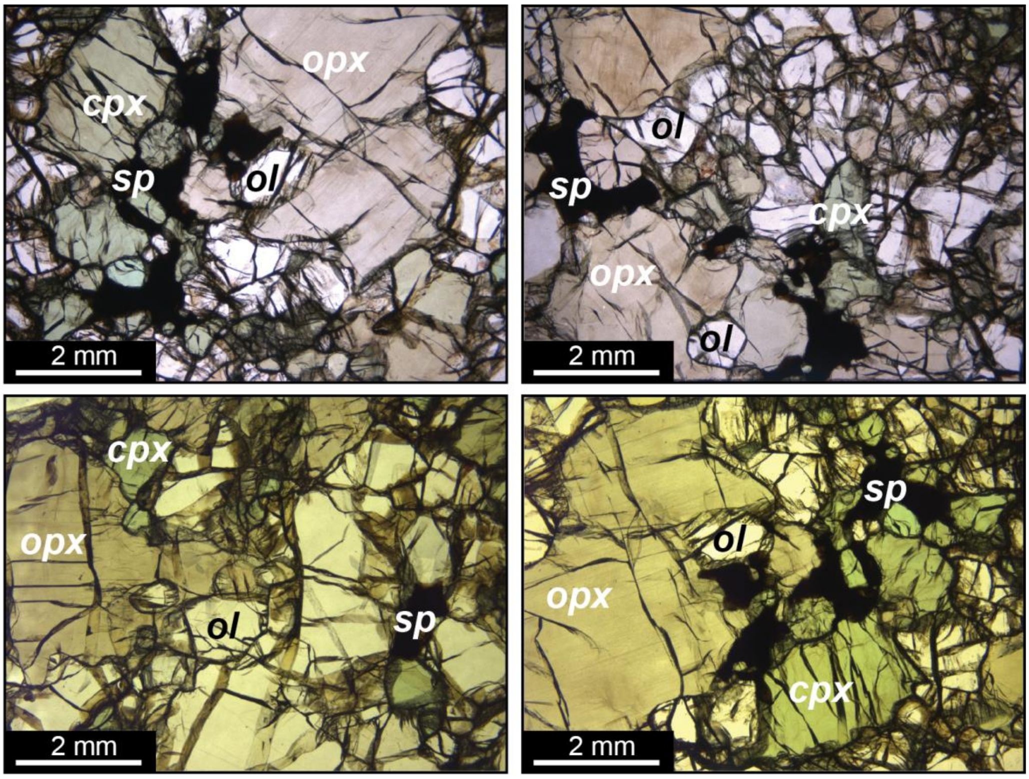

Figure S3. Photomicrographs of transmitted light optical microscope of representative thin section samples of protogranular mantle xenoliths from Coyhaique. Abbreviations: ol – olivine; opx – orthopyroxene; cpx – clinopyroxene; sp – spinel.

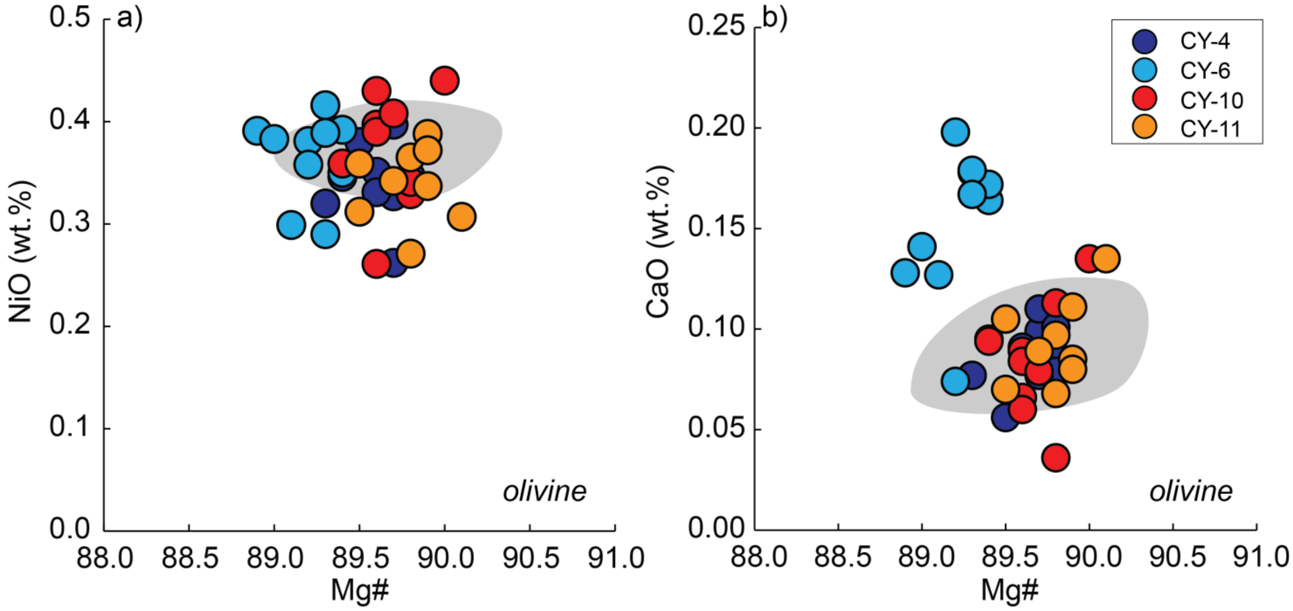

Figure S4. Co-variation diagrams of Mg# [100 x $Mg^{2+}/(Mg^{2+}+Fe^{2+})$] versus NiO (a) and CaO (b) contents (wt.%) in olivine from Coyhaique peridotite xenoliths, compared with previously published data from the same locality (grey fields)[11].

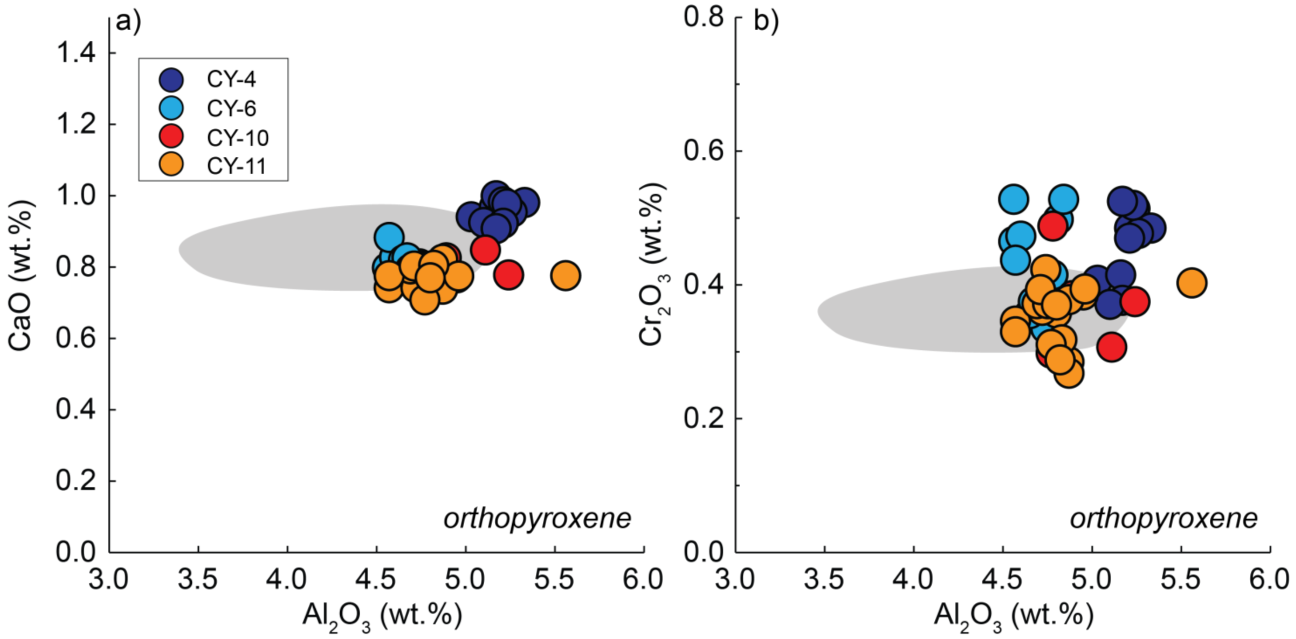

Figure S5. Co-variation diagrams of Al_2O_3 versus CaO (a) and Cr_2O_3 (b) contents (wt.%) in orthopyroxene from Coyhaique peridotite xenoliths, compared with previously published data from the same locality (grey fields)[11].

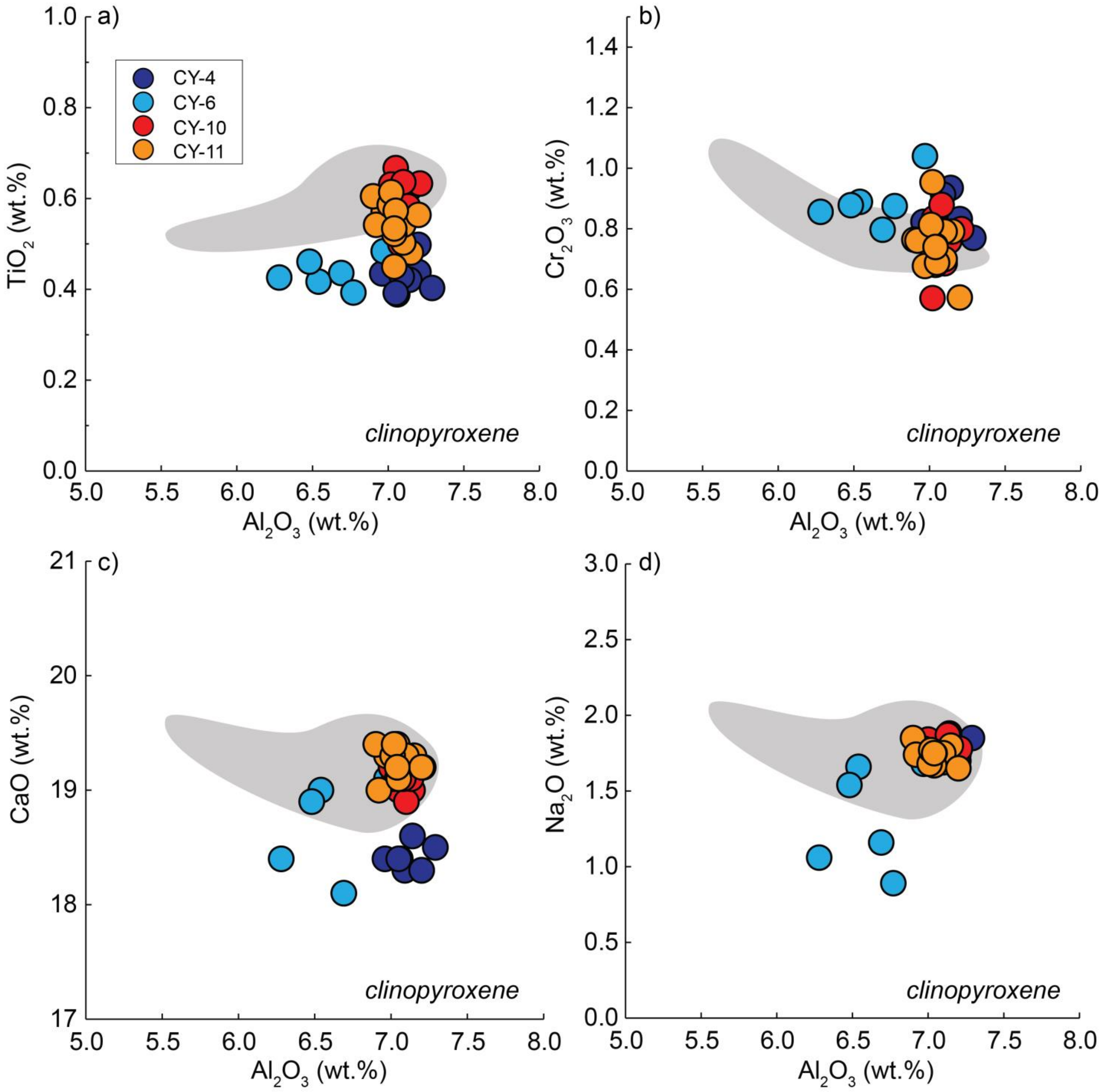

Figure S6. Co-variation diagrams of Al_2O_3 versus TiO_2 (a), Cr_2O_3 (b), CaO (c) and Na_2O (d) contents (wt.%) in clinopyroxene from Coyhaique peridotite xenoliths, compared with previously published data from the same locality (grey fields)[11].

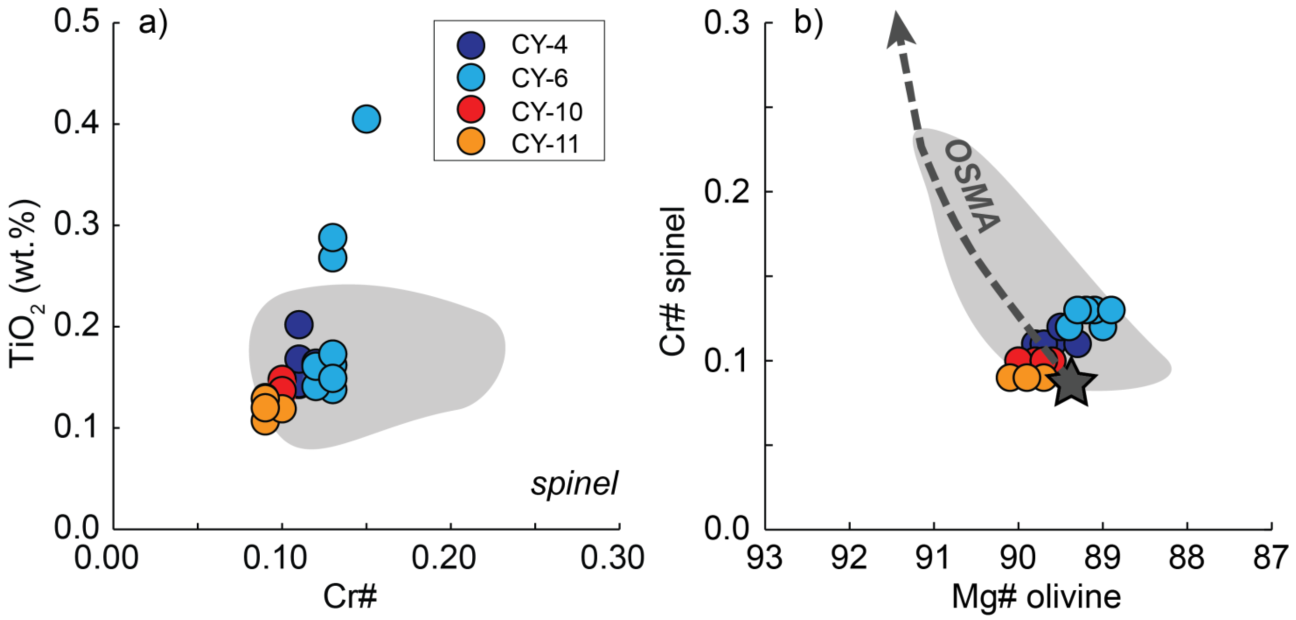

Figure S7. Co-variation diagrams of Cr# [$Cr^{3+}/(Cr^{3+}+Al^{3+}+Fe^{3+})$] and TiO_2 abundances (wt.%) in spinel from Coyhaique peridotite xenoliths, and of olivine Mg# [100 x Mg/(Mg + Fe^{2+})] versus co-existing spinel Cr# (b) compared with olivine-spinel mantle array (OSMA)[15] and previously published data from the same locality (grey fields)[11]. Grey star refers to the composition of fertile MORB mantle[16].

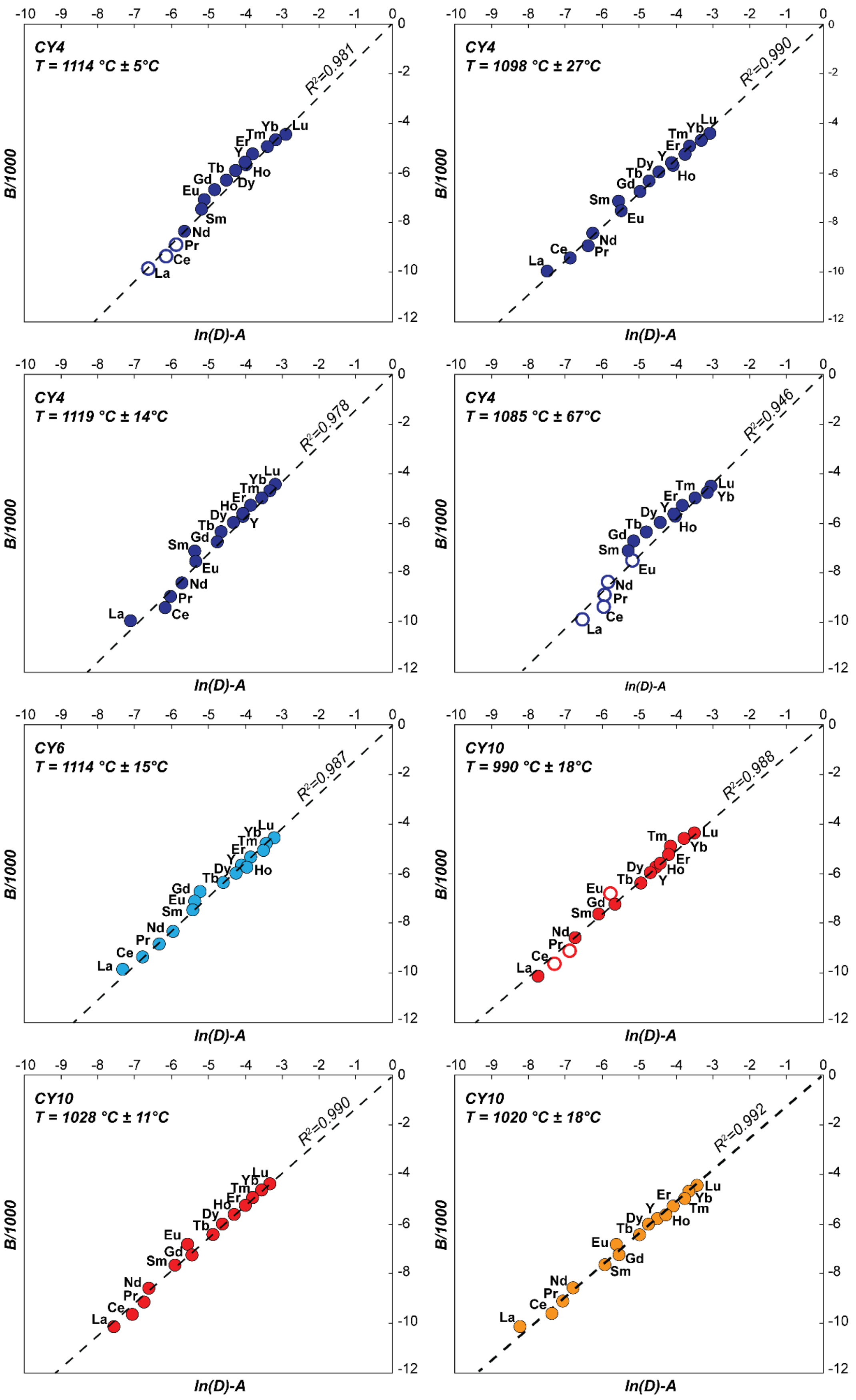

Figure S8. Inversion diagrams used to calculate temperature conditions in Coyhaique mantle xenoliths based on the REE-in-two-pyroxene thermometer[17]. Temperature conditions are inverted through least square linear fit (dashed lines) to REE data obtained after excluding the outliers (empty circles).

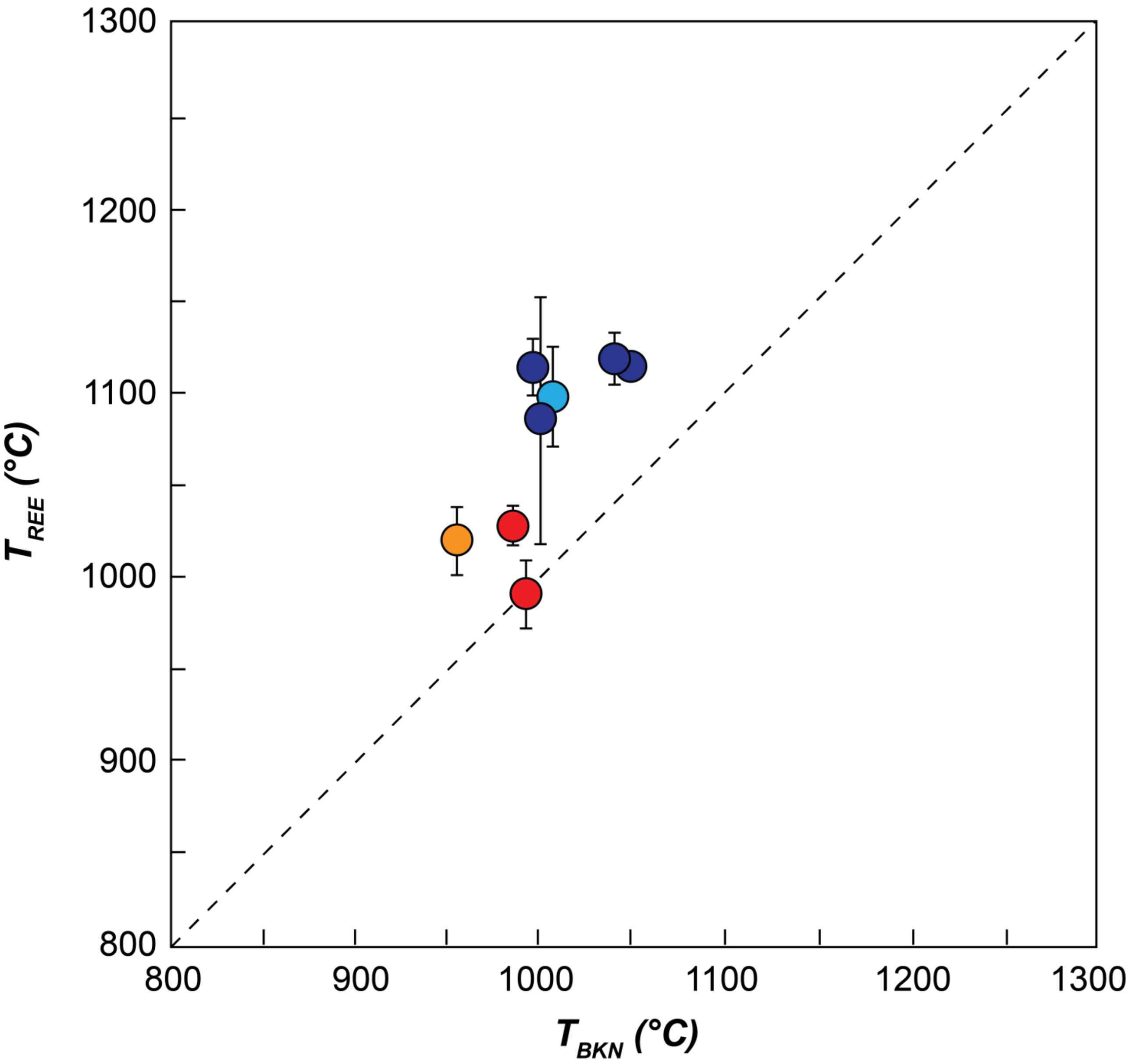

Figure S9. Comparison of temperature conditions calculated with REE-in-two-pyroxenes[17] and those calculated with Ca-in-opx thermometers[18] in Coyhaique mantle xenoliths, relative to a 1:1 correlation line (dashed line).
